# The proliferation and invasion of osteosarcoma are inhibited by miR-101 via targetting ZEB2

**DOI:** 10.1042/BSR20181283

**Published:** 2019-02-08

**Authors:** Haopeng Lin, Xiaodong Zheng, Ting Lu, Yang Gu, Canhao Zheng, Huajie Yan

**Affiliations:** 1 Department of Orthopedics, Shantou Chaonan Minsheng Hospital, Shantou, China; 2 Department of Orthopedics, Chaoyang Distric, Shantou City, Dafeng Hospital, Shantou, China; 3 Breast Tumor Center, Sun Yat-sen Memorial Hospital, Sun Yat-sen University, Guangzhou, China; 4 Department of Emergency, Sun Yat-sen Memorial Hospital, Sun Yat-sen University, Guangzhou, China

**Keywords:** miR-101, osteosarcoma, progression, ZEB2

## Abstract

Having a better grasp of the molecular mechanisms underlying carcinogenesis and progression in osteosarcoma would be helpful to find novel therapeutic targets. Different types of cancers have presented abnormal expression of miRNA-101 (miR-101). Nevertheless, we still could not figure out what expression of miR-101 in human osteosarcoma is and its biological function. Thus, we conducted the present study to identify its expression, function, and molecular mechanism in osteosarcoma. We detected the expression of miR-101 in osteosarcoma samples and cell lines. The effects of miR-101 on osteosarcoma cells’ proliferation and invasion were evaluated. Luciferase reporter assay was applied to identify the direct target of miR-101. Compared with adjacent normal specimens and normal bone cell line by using qPCR, the expression levels of miR-101 in osteosarcoma specimens and human osteosarcoma cell lines distinctly decreased. According to function assays, we found that overexpression of miR-101 significantly inhibited the cell proliferation and invasion in osteosarcoma cells. Moreover, we confirmed that zinc finger E-box binding homeobox 2 (ZEB2) was a direct target of miR-101. In addition, overexpression of ZEB2 could rescue the inhibition effect of proliferation and invasion induced by miR-101 in osteosarcoma cells. MiR-101 has been proved to be down-regulated in osteosarcoma and has the ability to suppress osteosarcoma cell proliferation and invasion by directly targetting ZEB2.

## Introduction

Osteosarcoma is one of the most common primary malignant bone tumors and it accounts for approximately one-fifth of all primary bone cancers worldwide [1,2]. Children and young adults are the susceptible population and osteosarcoma brings serious public health problems and significant economic burden [3]. Even though the diagnostic and treatment methods including computed tomography, chemotherapy, and surgery have improved prognosis of patients a lot in recent years, local recurrence occurs in approximately 10% of patients and the survival rate is only 10% in metastatic or relapse patients [4–6]. Therefore, it is urgent to excavate the molecular mechanisms for osteosarcoma progression and discover the potential molecular markers for tumor-targetted therapy.

As a series of small and highly conserved RNA, microRNA (miRNA) contains 18–25 nts in length and is known for activating or inhibiting the progression of various cancers. MiRNA has also been proposed as a novel target for anticancer therapy in recent years [7,8]. By means of directly targetting oncogenes or tumor suppressor genes, no less than 50% of miRNAs are found to be involved in human tumorigenesis [9,10]. For example, miR-451 inhibits tumor growth, migration, and angiogenesis by way of directly targetting IL-6R in osteosarcoma [11]. Serving as an oncogene, *miR-27a* is able to promote osteosarcoma cells proliferation, migration, and invasion via MAP2K4 [12]. From several recent studies, we have learnt that miR-101 is significantly down-regulated and mainly serves as a tumor suppressor in various human cancers. However, one specific miRNA might act in different roles including oncogene or tumor suppressor depending on different tissues and environments. The expression pattern, biological roles, and potential molecular mechanism of miR-101 in the osteosarcoma have not been discovered yet.

The first step of the present study was to detect the expression level of miR-101 in osteosarcoma tissues and cell lines. Second, we continued to explore the biological function of miR-101 in osteosarcoma cells’ phenotype. In the last step, zinc finger E-box binding homeobox 2 (ZEB2) was verified as a direct target of miR-101. The results demonstrated that the miR-101/ZEB2 axis may be a promising therapeutic strategy for osteosarcoma treatment in the future.

## Materials and methods

### Clinical tissues and cell lines

We collected human osteosarcoma specimens and their adjacent normal tissues (30 pairs) from patients who underwent surgery. The human protocol was approved by the Ethics Review Board of the Shantou Chaonan Minsheng Hospital. Informed consent has been obtained from every patient. We purchased the osteosarcoma cell lines (U2OS, G292, MG63, SJSA2 and KHOS) and human osteoblast cell line HOB-c from the ATCC (Manassas, VA, U.S.A.). All the cells were maintained in Dulbecco’s modified Eagle’s medium (DMEM, HyClone, Beijing, China) with FBS (Thermo Scientific, Grand Island, NY, U.S.A.). and incubated in a humidified atmosphere with 5% CO_2_ and humidified sphere of 95% at 37°C.

### Cell transfection

We obtained miR-101 mimic and miR-101 negative control from GenePharma (Shanghai, China), then termed miR-101 mimic as miR-101 and miR-101 negative control as miR-NC in a convenient way. The pcDNA3.1s including ZEB2-pcDNA3.1 (pc-ZEB2) and negative control pcDNA3.1 (pc-NC) were purchased from Ribobio (Guangzhou, China). In cell transfection, which was performed with Lipofectamine 2000 Reagent (Invitrogen, Carlsbad, New Mexico, U.S.A.) according to the manufacturer’s instructions, we seeded cells in six-well plates and cultured them until 60–75% confluency was reached.

### Transwell assays

The invasion ability of osteosarcoma cells was accessed with transwell chambers (Corning Costar, MA, U.S.A.). For the invasion assays, 100 μg of Matrigel was used to coat the transwell inserts (BD, NJ, U.S.A.) and 3 × 10^4^ cells resuspended in 0.1 ml of serum-free DMEM were added into it. Then DMEM with 10% FBS was added to the bottom wells. After incubation for 36 h, we removed the cells on the upper surface of the membrane, fixed the cells on the lower surface with methanol, stained them with 0.1% Crystal Violet, and then counted them under a light microscope.

### Cell count kit-8

Cell count kit-8 (CCK8) assays were performed to investigate the proliferation of osteosarcoma cells. We seeded cells into 96-well plates (2 × 10^3^/well) and added 10 μl CCK8 reagent to each well at a fixed time point each day. After incubation for 4 h, the absorbance of each well at 450 nm was measured with a microplate reader.

### Colony formation assay

A total of 500 cells were seeded in six-well plates individually in the colony formation assay and after 14 days of cultivation, the cells were washed with PBS, fixed, and stained with Giemsa, then counted the clone number (cells population > 50) with a microscope.

### Dual luciferase assay

Binding sequences were predicted by TargetScan7.2. The fragment of the ZEB2 3′-UTR that contains the miR-101 binding site was synthesized and deemed as wild-type ZEB2 (wt-ZEB2). The mutant fragment of the ZEB2 3′-UTR that do not contain the miR-101 binding site was also synthesized and regarded as mutant ZEB2 (mt-ZEB2). Then the sequences cloned into psiCHECK-2 vector (Promega, Madison, WI, U.S.A.). We co-transfected the osteosarcoma cells with former luciferase reporter vector with or without miR-101 mimic and measured luciferase activity with the help of Dual-Luciferase Reporter Assay System (Promega, Madison, WI, U.S.A.). *Renilla* luciferase activity was used for control when normalizing results.

### Total RNA extraction and qPCR

In accordance with the protocol of RNA extraction, we dissolved the cells in TRIzol reagent (Invitrogen, Carlsbad, New Mexico, U.S.A.) in order to extract the total RNA. After spectrophotometric quantitation, we synthesized cDNA with iScript cDNA Synthesis Kit. qPCR was performed with the help of SYBR Premix ExTaq (Takara Biotechnology, Osaka, Japan). β-Actin and U6 snRNA played a role of internal controls for detection. The primers used are listed as follows: miR-101, forward 5′-CGCCGATCGATCGATTCTG-3′, reverse 5′-CGATCATTTTTTTTTTTTTTTGAC-3′; U6, forward 5′-CTTCGGCAGCACATATAC-3′, reverse 5′-TTCACGAATTTGCGTGTCAT-3′; ZEB2, forward 5′-CAAGAGGCGCAAACAAGCC-3′, reverse 5′-GGTTGGCAATACCGTCATCC-3′; β-Actin, forward 5′-TCCCTGGAGAAGAGCTACG-3′, reverse 5′-GTAGTTTCGTGGATGCCACA-3′. We calculated the relative expression level of miR-101 and ZEB2 using the 2^−ΔΔ*C*^_T_ method and normalized it to the control.

### Western blot

A total of 25 μg proteins from the lysates of the osteosarcoma cells were added to electrophoresis through SDS/PAGE and then transferred them on to PVDF membranes. Then, these membranes were incubated with primary antibodies for ZEB2 (Abcam, Cambridge, MA, U.S.A.) or β-actin (Beyotime, Nantong, China) overnight at 4°C, and with horseradish peroxidase–conjugated goat anti-rabbit antibody (Santa Cruz Biotechnology, Santa Cruz, CA, U.S.A.) at room temperature for 2 h, respectively. Finally, the signals were detected by ECL Plus (Beyotime, Nantong, China) as per the manufacturer’s instructions and then calculated the relative protein levels with β-actin as the loading control.

### Statistical analysis

Descriptive statistics including percentages or medians were used to summarize patients’ characteristics. Student’s *t* test was conducted to compare the difference between two groups for continuous measures. Chi-square test was performed to compare the difference for discontinuous measures. ANOVA was applied to examine the differences amongst three or more groups for normal distribution data and once the significant difference was confirmed, then Dunnett’s test was used to detect the difference in any two groups from them. Statistical significance was set at *P*<0.05. All statistical analyses were performed using SPSS 13.0.

## Results

### MiR-101 was obviously down-regulated in osteosarcoma tissues and cell lines

**Figure 1:**
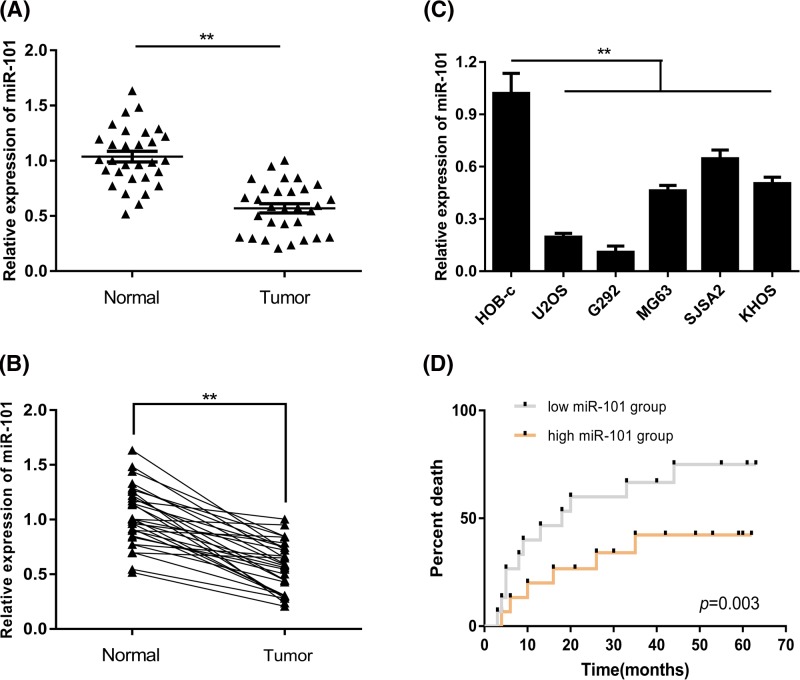
miR-101 was significantly down-regulated in osteosarcoma (**A,B**) Compared with normal tissue, miR-101 was down-regulated in osteosarcoma tissues. (**C**) The expression of miR-101 was lower in osteosarcoma cell lines than normal osteoblast cell line. (**D**) Survival analysis found patients with higher miR-101 expression had better survival than patients with lower expression; (**P<0.01).

A total of 30 pairs of osteosarcoma specimens and adjacent normal tissues were collected for the detection of miR-101. According to the results, we found that miR-101 was significantly down-regulated in osteosarcoma tissues compared with adjacent normal tissues (Figure 1A,B). Complying with clinical specimens, miR-101 was also significantly down-regulated in osteosarcoma cell lines compared with human osteoblast cell line HOB-c (Figure 1C). Moreover, the patients were divided into two groups based on their miR-101 expression to perform survival analysis (median follow-up time: 28 months). It was found that patients with higher miR-101 expression had better survival quality than patients with lower expression (Figure 1D). The clinical information of the enrolled patients is presented in Table 1. It was also found that the miR-101 expression level was correlated with tumor stage and metastatic status.

**Table 1:** The relation between clinical information of patients and miR-101 level

Clinicopathological status	miR-101 high (number of patients)	miR-101 low (number of patients)	*P*-value
Age (years)			0.70
>18	8	8	
≤18	8	6	
Gender			0.58
Male	7	8	
Female	9	6	
Living region			0.79
City	10	7	
Country	7	6	
Location			0.88
Femur/tibia	14	12	
Elsewhere	2	2	
T stage			<0.01
I–II	12	5	
III–IV	2	11	
Metastasis			<0.01
Yes	2	10	
No	15	3	

### The proliferation and invasion of osteosarcoma cell was inhibited by miR-101

**Figure 2:**
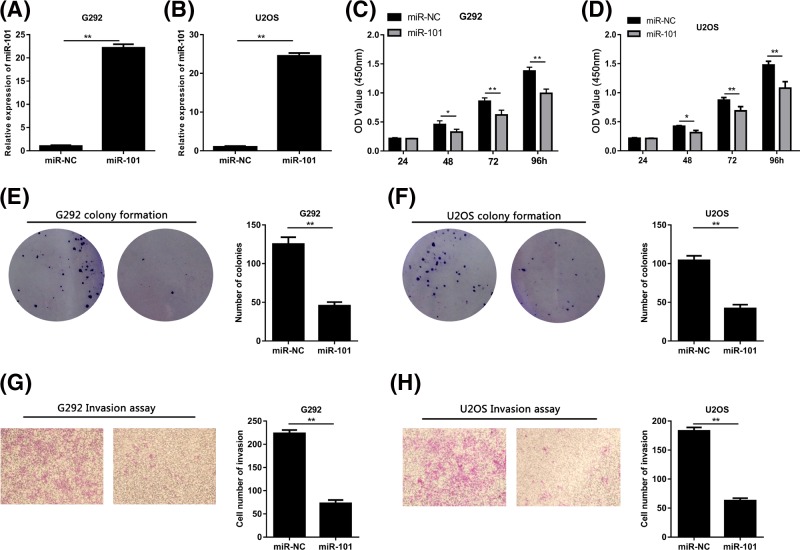
Osteosarcoma cell growth and invasion were inhibited by miR-101 (**A,B**) Transfection of miR-101 mimic increased the expression of miR-101 in G292 and U2OS cells. (**C,D**) CCK8 assays revealed that miR-101 significantly inhibited the cell viability. (**E,F**). Results of colony formation assays indicated that cell viability was depressed by miR-101. (**G,H**) Transwell assays showed that cell invasion was suppressed by miR-10 (*P<0.05, **P<0.01).

To investigate the biological functions of miR-101 in osteosarcoma, we transfected the G292 and U2OS cells with miR-101 (Figure 2A,B). According to CCK8 assay results, it was clear that the cell viability significantly decreased in osteosarcoma cells transfected miR-101 mimic compared with osteosarcoma cells transfected miR-NC (Figure 2C,D). In accordance with these results, the colony formation was remarkably inhibited by overexpression of miR-101 in osteosarcoma cells by transfection of miR-101 mimic (Figure 2E,F). We then conducted invasion analyses for G292 and U2OS cells transfected with miR-101 or miR-NC by transwell assays to explore the effects of miR-101 on the metastasis ability of osteosarcoma cells. It was proved that invasion ability in osteosarcoma cells have greatly decreased by miR-101 restoration (Figure 2G,H).

### MiR-101 directly targetted ZEB2 in osteosarcoma cells

**Figure 3:**
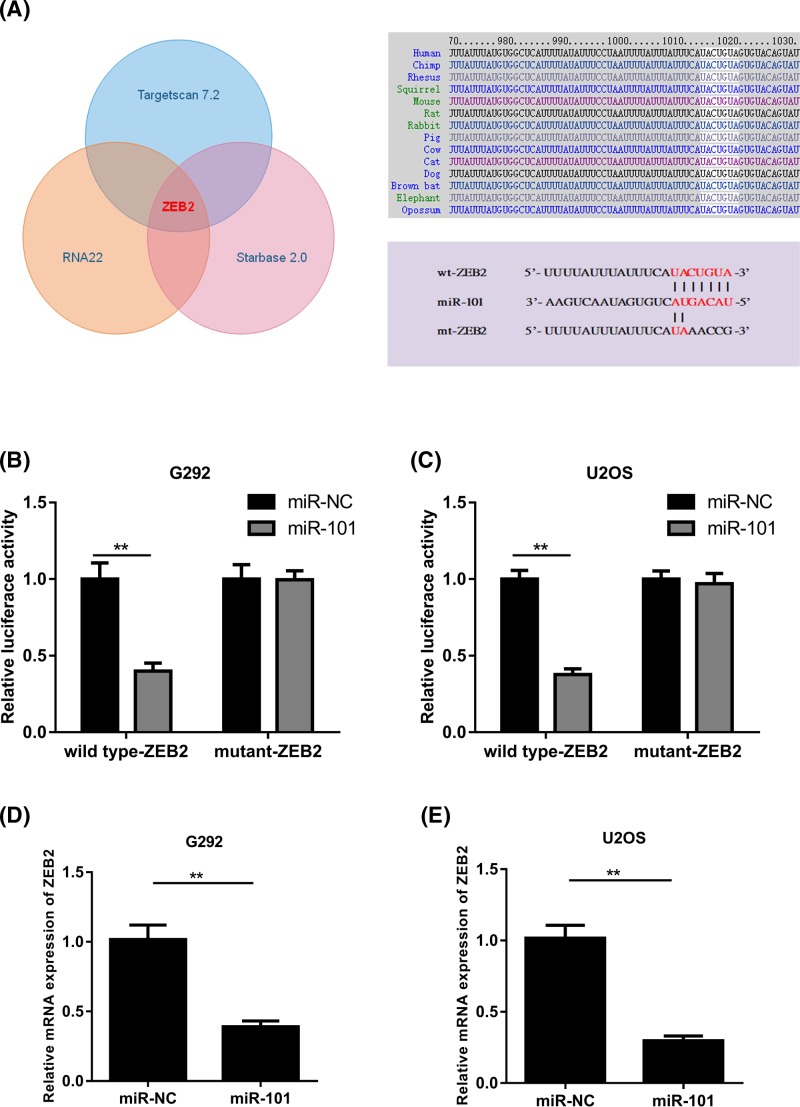
ZEB2 is identified as a direct target of miR-101 (**A**) The predicted binding sequence between human miR-101 and the 3′-UTR of ZEB2. (**B,C**) Luciferase assay results for G292 and U2OS cells showed that luciferase activity was decreased by miR-101 up-regulation in wild-type groups but not in mutant groups. (**D,E**) Results of qPCR assays revealed that the expression levels of ZEB2 in G292 and U2OS cells was inhibited by miR–101 (**P<0.01).

To vividly show the underlying molecular mechanisms that how miR-101 suppresses osteosarcoma cancer progression, potential targets of miR-101 were searched using TargetScan 7.2, RNA22, and Starbase 2.0. Amongst the candidates, *ZEB2*, an oncogene gene that plays an important role in various cancers, was predicted to be an miR-101 target and was selected for further experimental verification. We displayed the predicted interaction between miR-101 and the target site in the ZEB2 3′-UTR in Figure 3A. Then luciferase reporter assays were performed to explore whether miR–101 targets ZEB2 by binding to its 3′–UTR or not. The G292 and U2OS cells were co-transfected with the wild-type ZEB2 3′–UTR or mutant ZEB2 3′–UTR reporter vector and miR-101 mimics or miR–NC. According to the results, it was clear that luciferase activities significantly decreased only in the G292 and U2OS cells transfected with the wild-type ZEB2 reporter vector (Figure 3B,C) but the same result was not found in the cells with the mutant ZEB2 reporter vector. Furthermore, qPCR assays showed that miR–101 overexpression decreased the mRNA expression of ZEB2 in G292 and U2OS cells (Figure 3D,E), which illustrated that miR–101 directly targetted ZEB2 by binding to its 3′–UTR region in osteosarcoma cells.

### ZEB2 was up-regulated in osteosarcoma and related to prognosis

**Figure 4:**
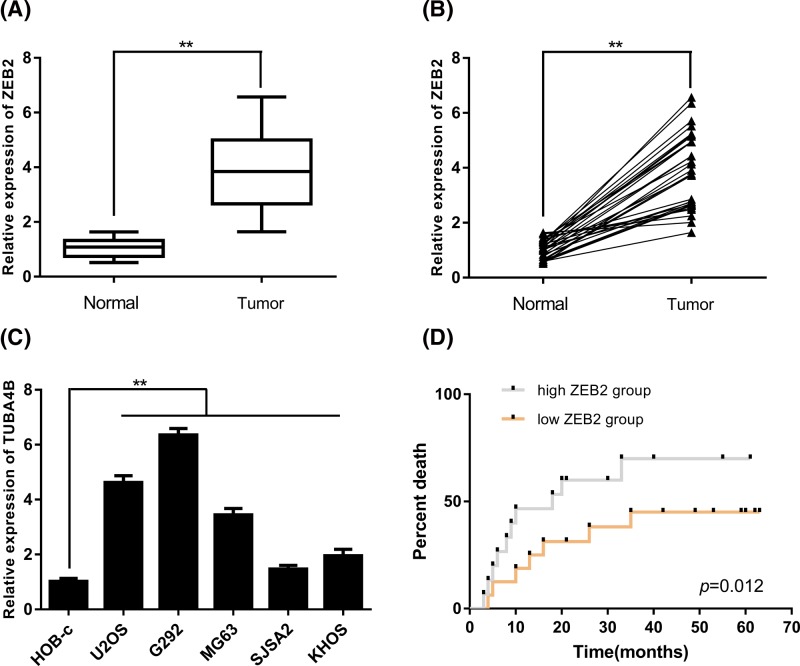
ZEB2 was up-regulated in osteosarcoma and correlated with prognosis (**A,B**) Compared with adjacent normal tissues, ZEB2 was up-regulated in osteosarcoma tissues. (**C**) Compared with normal tissues, ZEB2 was significantly up-regulated in osteosarcoma cell lines than normal human osteoblast cell line. (**D**) Patients with high ZEB2 expression had poorer prognosis than patients with low ZEB2 expression (**P<0.01).

The expression of ZEB2 in osteosarcoma tissues and adjacent normal tissues were then detected using qPCR assays. As the results showed, it was found that ZEB2 was significantly up-regulated in osteosarcoma tissues when compared with the adjacent normal tissues (Figure 4A,B). Moreover, we collected the RNA from normal human osteoblast cell line HOB-c and osteosarcoma cell lines (including U2OS, G292, MG63, SJSA2, and KHOS). The expression of *ZEB2* mRNA was significantly higher in osteosarcoma cell lines (including U2OS, G292, MG63, SJSA2, and KHOS) than those from normal human osteoblast cell line HOB-c (Figure 4C). In addition, the former patients were classified into two groups based on their ZEB2 expression. Results conducted by survival analysis showed that patients with high ZEB2 expression had poorer prognosis than those with low ZEB2 expression (Figure 4D). These results illustrated that ZEB2 was up-regulated in osteosarcoma and related to prognosis of osteosarcoma patients.

### The osteosarcoma progression was suppressed by miR-101 via ZEB2

**Figure 5:**
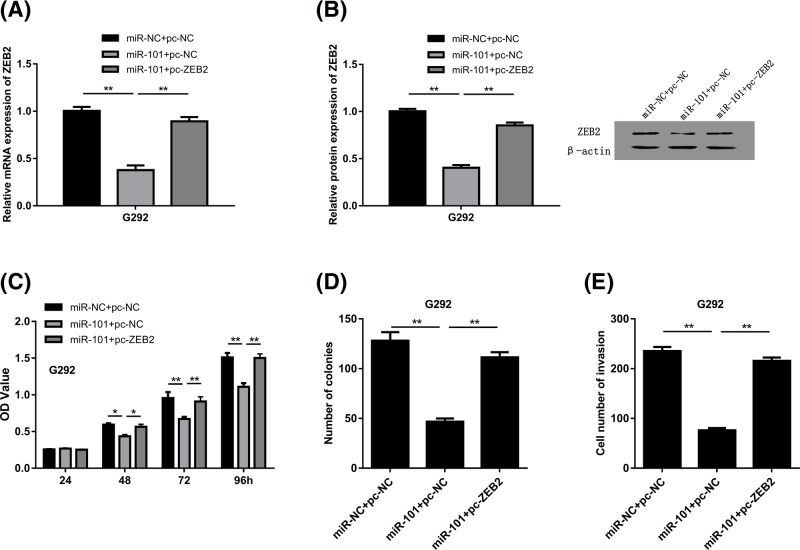
ZEB2 mediated the suppressive effects of miR-101 on osteosarcoma (**A**) The mRNA expression level of ZEB2 was inhibited by miR-101 but reversed by ZEB2. (**B**) Western blot assays revealed that the protein of ZEB2 was inhibited by miR-101 but reversed by ZEB2. (**C–E**) Phenotype rescue experiments suggested that the functions of miR-101 were mediated by ZEB2 (*P<0.05, **P<0.01).

To explore the potential functions of ZEB2 in osteosarcoma, we co-transfected the G292 cells with pc-ZEB2 or miR-101. As presented in Figure 5A, the mRNA expression level of ZEB2 was inhibited by miR-101 but reversed by pc-ZEB2. Western blot assays demonstrated the similar results (Figure 5B). From CCK8 assay and colony formation results, it was also noticed that the inhibition of cell proliferation by miR-101 could be reversed by ZEB2 overexpression (Figure 5C,D). Moreover, from the transwell assays, it is confirmed that the inhibition of cell invasion by miR-101 was greatly rescued by ZEB2 overexpression in osteosarcoma cell (Figure 5E).

## Discussion

From previous studies, it was safe to draw a conclusion that the abnormal expression of miR-101 was closely related to lung cancer, osteosarcoma, glioblastoma, and endometrial carcinoma [13–16]. For example, it was found that miR-101 was significantly down-regulated in cervical cancer patients’ serum when compared with healthy control group. Moreover, they noticed that the miR-101 expression was associated with clinical stage, lymph node metastasis, and overall survival of cervical cancer patients [17]. It was also reported that miR-101 was significantly underexpressed in gastric cancer tissues than that in normal gastric mucosa. The miR-101 in plasma was remarkably underexpressed in gastric cancer patients compared with healthy ones and its expression was obviously correlated with the clinical stage, metastatic status, and prognosis of gastric cancer patients [18]. However, the expression of miR-101 in osteosarcoma was unknown. Amongst all the existing studies, we first detected miR-101 in osteosarcoma tissues and cell lines. The detection illustrated that miR-101 was significantly down-regulated in osteosarcoma tissues and cell lines when compared with normal tissues and normal cell line. These results were consistent with the previous researches and indicated that miR-101 might act as a tumor suppressor in osteosarcoma.

Former researches also tried to uncover the biological role of miR-101 in cancer cells and explore its phenotypes. Li et al. [19] found that miR-101 could inhibit breast cancer cell proliferation, invasion, tumor growth, and lung metastasis. The research of Liu et al. [16] demonstrated that miR-101 significantly reduced in endometrial carcinoma and it negatively regulated angiogenesis and tumor growth of endometrial carcinoma. We explored the biological effects of miR-101 on osteosarcoma. We discovered that the proliferation and invasion of osteosarcoma cell lines were largely suppressed by the up-regulation of miR-101 and it might be the first study to illustrate the phenotype of miR-101 on osteosarcoma. Moreover, most miRNAs exert their functions by inversely regulating their target genes. The direct targets of miR-101 that have been ascertained include sex-determining region SRY-box9 protein (SOX9) [20], ubiquitin-specific protease 22 (USP22) [21], glycogen synthase kinase 3β (GSK3β) [22], and cyclin-dependent kinase 8 (CDK8) [23]. Using dual luciferase assays, we firstly identified a new target of miR-101, ZEB2, in the present study. ZEB2 is an important member of the ZEB family of two-handed zinc-finger/homeodomain proteins [24]. ZEB2 has been found to be a transcriptional repressor of E-cadherin and could promote the epithelial–mesenchymal transition, which contribute to the tumor metastasis and progression [25]. It also has been identified that ZEB2 could be up-regulated in various types of human cancers and acts as a potential oncogene in breast cancer and gastric cancer. Our studies illustrated that miR-101 suppressed osteosarcoma progression via ZEB2, which contributed a better understanding of ZEB2 on osteosarcoma. Future studies should be focussed on exploring the miR-101/ZEB2 functions *in vivo* and excavating their downstream pathways.

In conclusion, miR-101 has been found to be greatly down-regulated in osteosarcoma. Furthermore, it is demonstrated, for the first time, that the miR-101/ZEB2 axis plays an essential role in regulating osteosarcoma proliferation and metastasis. The newly identified miR-101/ZEB2 link offers a new insight into the mechanisms underlying osteosarcoma development, and provides a promising therapeutic strategy for osteosarcoma treatment in the future.

## Abbreviations

CCK8: cell count kit-8
DMEM: Dulbecco’s modified Eagle’s medium
pc-ZEB2: ZEB2-pcDNA3.1
ZEB2: zinc finger E-box binding homeobox 2
